# Clinical phenotype and prognosis of real‐world patients with wild‐type transthyretin amyloid cardiomyopathy treated with tafamidis

**DOI:** 10.1002/ejhf.70071

**Published:** 2025-11-29

**Authors:** Aldostefano Porcari, Paolo Milani, Simone Longhi, Francesco Cappelli, Fabio Vagnarelli, Alberto Aimo, Alberto Cipriani, Elisa Gardini, Stefania Marazia, Emanuele Monda, Giacomo Tini, Beatrice Musumeci, Matteo Serenelli, Anna Cantone, Carla Lofiego, Marco Marini, Giuseppe Vergaro, Grazia Foti, Francesco Musca, Daniela Tomasoni, Giacomo Bonacchi, Federica Colio, Giulio Sinigiani, Laura De Michieli, Francesca Sturdà, Marco Pozzan, Piero Gentile, Samuela Carigi, Michela Bartolotti, Giuseppe Sena, Irene Ruotolo, Giuseppe Damiano Sanna, Margherita Zanoletti, Marco Canepa, Massimo Di Marco, Emilia D'Elia, Gianluca Di Bella, Mauro Driussi, Massimo Imazio, Federico Perfetto, Elena Biagini, Giuseppe Limongelli, Marco Metra, Michele Emdin, Giampaolo Merlini, Stefano Perlini, Marco Merlo, Giovanni Palladini, Gianfranco Sinagra

**Affiliations:** ^1^ Center for Diagnosis and Treatment of Cardiomyopathies, Cardiovascular Department, Azienda Sanitaria Universitaria Giuliano‐Isontina (ASUGI), University of Trieste Trieste Italy; ^2^ European Reference Network for Rare, Low Prevalence and Complex Diseases of the Heart ERN GUARD‐Heart Trieste Italy; ^3^ National Amyloidosis Centre, Division of Medicine, University College London, Royal Free Hospital London UK; ^4^ Department of Molecular Medicine, University of Pavia Pavia Italy; ^5^ Amyloidosis Research and Treatment Center, Fondazione IRCCS Policlinico San Matteo Pavia Italy; ^6^ Cardiology Unit, Cardiac Thoracic and Vascular Department, IRCCS Azienda Ospedaliero‐Universitaria di Bologna Bologna Italy; ^7^ Cardiomyopathy Unit, Careggi University Hospital, University of Florence Florence Italy; ^8^ Tuscan Regional Amyloidosis Centre, Careggi University Hospital Florence Italy; ^9^ Cardiology Unit, ‘G.M. Lancisi’ Cardiovascular Center, Azienda Ospedaliero Universitaria delle Marche Ancona Italy; ^10^ Interdisciplinary Center of Health Sciences, Scuola Superiore Sant'Anna, Fondazione Toscana Gabriele Monasterio Pisa Italy; ^11^ Cardiology Division, Fondazione Toscana Gabriele Monasterio Pisa Italy; ^12^ Department of Cardiac, Thoracic and Vascular Sciences and Public Health, University of Padua Padua Italy; ^13^ Cardiology Unit, Morgagni‐Pierantoni Hospital Forlì Italy; ^14^ Cardiology Division, Vito Fazzi Hospital Lecce Italy; ^15^ Inherited and Rare Cardiovascular Disease Unit, University of Campania ‘Luigi Vanvitelli’, AORN dei Colli, Monaldi Hospital Naples Italy; ^16^ Department of Clinical and Molecular Medicine Faculty of Medicine and Psychology, Sapienza University Rome Italy; ^17^ Cardiologic Centre, University of Ferrara Cona (FE) Italy; ^18^ Cardiology 4, ASST Niguarda Ca' Granda Hospital Milan Italy; ^19^ Cardiology, ASST Spedali Civili, Department of Medical and Surgical Specialities, Radiological Sciences, and Public Health, University of Brescia Brescia Italy; ^20^ Cardiology Unit, Infermi Hospital, AUSL della Romagna Rimini Italy; ^21^ Cardiology Unit, Bufalini Hospital, AUSL della Romagna Cesena Italy; ^22^ Department of Medical and Surgical Sciences DIMEC, Alma Mater Studiorum, University of Bologna Bologna Italy; ^23^ Clinical and Interventional Cardiology, Sassari University Hospital Sassari Italy; ^24^ Cardiovascular Unit, IRCCS Ospedale Policlinico San Martino Genoa Italy; ^25^ Department of Internal Medicine University of Genova Genoa Italy; ^26^ Cardiology Unit‐UTIC, Santo Spirito Hospital Pescara Italy; ^27^ Cardiovascular Department Papa Giovanni XXIII Hospital Bergamo Italy; ^28^ Department of Cardiology University of Messina Messina Italy; ^29^ Cardiology, Cardiothoracic Department Santa Maria della Misericordia Hospital Udine Italy; ^30^ Department of Internal Medicine University of Pavia Pavia Italy

**Keywords:** Transthyretin amyloid cardiomyopathy, Clinical phenotype, Disease‐modifying treatment, Survival

## Abstract

**Aims:**

Tafamidis reshaped the treatment paradigm in transthyretin amyloid cardiomyopathy (ATTR‐CM) based on a phase‐3 randomized controlled trial, but real‐world data on its use remain limited. This study aimed to assess in a large, contemporary, real‐world cohort of patients with wild‐type ATTR‐CM (ATTRwt‐CM) (i) the clinical phenotype of patients receiving tafamidis, and (ii) the association of tafamidis with survival using propensity‐matched observational data.

**Methods and results:**

Data of patients diagnosed with ATTRwt‐CM (January 2017 to June 2023) from 19 Italian centres were analysed. A propensity score (PS) reflecting the likelihood of being treated with tafamidis for each patient was determined using four variables that were significantly different among the two groups: age, New York Heart Association (NYHA) class, National Amyloidosis Centre (NAC) stage and mineralocorticoid receptor antagonists (MRAs). The primary outcome was all‐cause mortality. The study comprised 1556 ATTRwt‐CM patients: 965 (62%) patients initiated on tafamidis by June 2023 and 591 (38%) patients never treated with disease‐modifying therapy. Tafamidis‐treated patients were older, exhibited a lower NYHA class and NAC stage, and were more often treated with MRAs compared to untreated patients. The PS‐matched cohort comprised 426 patients treated with tafamidis and 426 PS‐matched untreated patients (mean age 78.9 ± 5.0 years, 88.3% men, 12.9% in NYHA class III). Adequacy of matching was verified (standardized differences: <0.20 between groups). Over 25 months (interquartile range: 15–40), treatment with tafamidis was associated with lower rates of all‐cause mortality (hazard ratio 0.55, 95% confidence interval 0.39–0.77, *p* = 0.001) across the spectrum of NAC disease stages (*p*‐interaction = 0.94).

**Conclusions:**

In this large, contemporary, real‐world cohort of patients with ATTRwt‐CM, predominantly in NYHA class I or II, treatment with tafamidis was consistently associated with a significantly lower risk of all‐cause mortality.

## Introduction

Wild‐type transthyretin amyloid cardiomyopathy (ATTRwt‐CM) is caused by the progressive accumulation of insoluble amyloid fibrils formed by transthyretin (TTR) in the myocardial extracellular space.^1^ The median survival from diagnosis of untreated patients was reported to be approximately 2–6 years.^2^, ^3^, ^4^, ^5^

Currently, the only drug approved for the treatment of ATTR‐CM in Europe is tafamidis,^6^ that is bound by and increases the stability of circulating transthyretin (TTR) in its normal soluble form, thereby reducing its propensity to misfold and form ATTR amyloid fibrils. In a phase 3 placebo‐controlled trial^7^ (Transthyretin Amyloidosis Cardiomyopathy Clinical Trial [ATTR‐ ACT]), patients treated with tafamidis showed 30% lower risk of all‐cause mortality over 30 months of treatment compared to placebo. A long‐term extension study (NCT02791230) showed better survival with early (i.e. randomized to tafamidis) as opposed to late (i.e. randomized to placebo) tafamidis treatment.^8^, ^9^ However, the trial population was enrolled before the validation of a non‐invasive algorithm for diagnosing ATTR‐CM in 2016.^10^ Real‐world data on tafamidis use in ATTR‐CM patients are limited, with available cohorts exhibiting milder disease stages compared to ATTR‐ACT trial participants and characterized by significant heterogeneity in patient characteristics, small sample sizes and short observation periods.^11^, ^12^, ^13^, ^14^ A real‐world study^15^ from the Transthyretin Amyloidosis Outcomes Survey (THAOS) reported outcome data in a large cohort of ATTR‐CM patients treated with tafamidis, including both wild type and variant forms; however, the majority of data originated from US study sites, where the healthcare system differs significantly from Europe.

Based on the recently published robust and favourable clinical trial data, acoramidis, a TTR stabilizer, and vutrisiran, a gene silencer, are soon to be recommended in clinical practice as an additional disease‐modifying treatment option.^16^, ^17^ As these treatments become more accessible, the need for real‐world data to personalize treatment strategies has become increasingly important.

The aim of the study was to assess, in a large, multicentre, real‐world database of contemporary patients with ATTRwt‐CM across Italy (i) the clinical phenotype of ATTRwt‐CM patients receiving tafamidis, and (ii) the association of tafamidis with mortality using propensity‐matched observational data.

## Methods

**Figure 1:**
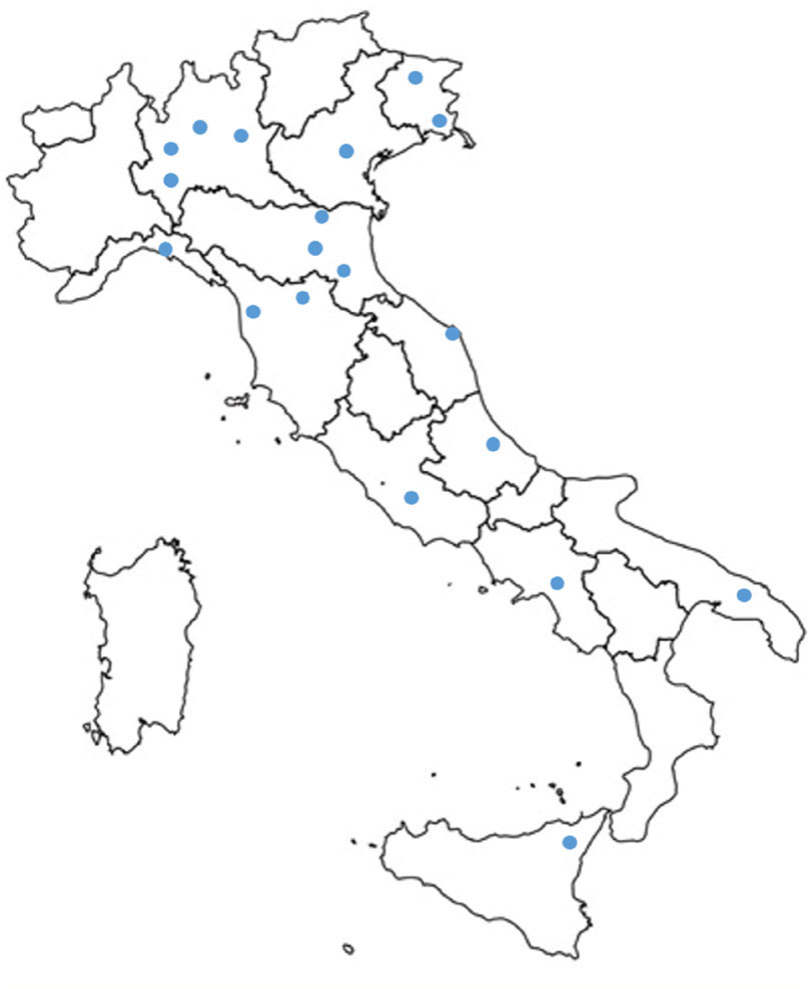
Participating centres across Italy. Blue dots identify the distribution of participating centres across Italy.

This is a multicentre, longitudinal, observational study performed across 19 centres in Italy, with contributions proportional to each site's caseload (*Figure* *1*, full list available in online supplementary *Table Appendix* *S1*). Local institutional review board approval for the study was obtained from each participating centre. The study was conducted according to the Declaration of Helsinki, and informed consent was obtained under the institutional review board policies of the relevant hospital administrations.

Patients diagnosed with ATTRwt‐CM from January 2017 (following development of a non‐invasive diagnostic algorithm for ATTR‐CM^10^) to June 2023 were included in the study, ensuring a contemporary cohort of patients identified under the same diagnostic paradigm with a minimum follow‐up observation period of 12 months.

Tafamidis has been approved for clinical use in Italy since October 2021 in patients with ATTR‐CM in New York Heart Association (NYHA) class I or II. Access to treatment for all patients with ATTR‐CM, including those in NYHA class III at diagnosis, was allowed under a compassionate use programme, which was activated 9 months before the commercial approval. All participating centres adhered to these nationally licensed indications.

Diagnosis of ATTR‐CM was established on the basis of a suggestive echocardiogram or cardiac magnetic resonance study and either endomyocardial biopsy proof of ATTR amyloid or Perugini grade 2 or 3 myocardial uptake on cardiac scintigraphy in the absence of both an abnormal serum free light‐chain ratio and a monoclonal component in the serum or urine by immunofixation.^10^ The National Amyloidosis Centre (NAC) ATTR staging was assessed at the time of diagnosis based on N‐terminal pro‐B‐type natriuretic peptide (NT‐proBNP) with a threshold of 3000 ng/L and estimated glomerular filtration rate (eGFR) with a cutoff of 45 ml/min/1.73 m^2^.^2^ As our patients had a combination of B‐type natriuretic peptide (BNP) and NT‐proBNP at baseline, the NAC ATTR stage was measured by applying a previously used conversion^18^ whereby a BNP cutoff >600 pg/ml corresponds to NT‐proBNP >3000 pg/ml. The *TTR* gene was sequenced in all patients as previously described.^19^ All echocardiographic parameters were measured according to current international standards (online supplementary material).^20^ All patients were enrolled into a protocolized follow‐up programme that consisted of 6 to 12 monthly consultations (or earlier according to individual clinical needs) based on participating centres' policy. Time 0 (the time each patient entered the study) was defined as the time of ATTRwt‐CM diagnosis.

### Outcome

The primary outcome of the study was all‐cause mortality. The mortality endpoint was defined as time to death from baseline for all deceased patients and time to censor date (30 June 2024) from baseline among the remainder.

### Statistical analysis

Descriptive statistics between the study groups were performed. All continuous variables were tested for normality (Shapiro–Wilk test) and are presented as mean ± standard deviation (SD) if the distribution was normal or median (Q1–Q3) otherwise. Categorical variables were expressed as absolute number and frequency (percentage). The two‐sample Student's *t*‐test for continuous variables was used to compare means if the data were normally distributed in each treatment group, and its non‐parametric equivalent, the Mann–Whitney U test, was used otherwise to compare the distributions of the two treatment groups. The chi‐square test or Fisher's exact test was used for categorical variables.

Propensity score (PS) matching was used to reduce confounding bias. The baseline characteristics of the unmatched population of 1556 ATTRwt‐CM patients (divided into two groups according to treatment) were assessed. After comparison between groups, four variables were found to be significantly different: age, NYHA class, NAC disease stage and treatment with mineralocorticoid receptor antagonists (MRAs) (*Table* *1*). A PS reflecting the likelihood of being treated with tafamidis for each patient was determined using the set of four variables. The level of balance between treatment and control groups was checked by visual analysis of a density plot of the distribution of the PS in the two groups after defining the area of common support and graph of the PS distribution within the area of common support. When plotting the distribution of PSs for the two matched groups using a diagram that showed the overlap between the two distributions (referred to as the area of common support), any additional patients lying outside this area were excluded. Patients were then matched on the basis of their PSs in the two medication groups in a 1:1 ratio using the nearest‐neighbour approach without replacement and caliper width equal to 0.20 times the SD of the logit of the PSs. Adequacy of matching was verified by ensuring that the standardized differences between groups were <0.20 for all variables used to create the PS.

**Table 1:** Baseline characteristics of the study population

Parameters	Tafamidis (*n* = 965)	Untreated (*n* = 591)	*p*‐value
Age, years	78.5 ± 6.5	77.8 ± 6.6	**<0.001**
Male sex	865 (89.6)	547 (92.6)	0.054
Atrial fibrillation	488 (50.6)	303 (51.3)	0.79
Ischaemic heart disease	201 (20.8)	119 (20.1)	0.74
Diabetes mellitus	161 (16.7)	110 (18.6)	0.33
Hypertension	655 (67.9)	402 (68.0)	0.95
Heart failure severity			
NYHA class			**<0.001**
I	171 (17.7)	78 (13.6)	
II	734 (76.1)	372 (64.8)	
≥III	60 (6.2)	124 (21.6)	
NAC stage			**<0.001**
1	583 (63.1)	216 (43.3)	
2	278 (30.1)	217 (43.5)	
3	63 (6.8)	66 (13.2)	
Echocardiographic parameters			
MWTd, mm	17.5 ± 2.9	17.6 ± 2.9	0.47
LVEF, %	51.9 ± 9.7	51.4 ± 10.8	0.61
Medications			
Beta‐blockers	543 (56.3)	341 (57.9)	0.53
ACEi/ARBs/ARNI	509 (52.7)	309 (52.3)	0.86
MRAs	384 (39.8)	169 (28.6)	**<0.001**

Values are presented as mean ± standard deviation, or *n* (%).

ACEi, angiotensin‐converting enzyme inhibitor; ARB, angiotensin receptor blocker; ARNI, angiotensin receptor–neprilysin inhibitor; LVEF, left ventricular ejection fraction; MRA, mineralocorticoid receptor antagonist; MWTd, maximal wall thickness in diastole; NAC, National Amyloidosis Centre; NYHA, New York Heart Association.

Survival analyses were performed in the overall study population and in the PS‐matched population. To avoid the ‘immortal time’ bias, the association between tafamidis treatment and all‐cause mortality was assessed using a time‐dependent Cox regression analysis with tafamidis use as time‐varying exposure. In this analysis, each individual not treated with tafamidis within 3 months of ATTR‐CM diagnosis is identified as ‘not on treatment’ from time 0 (i.e. ATTR‐CM diagnosis) to the day before initiation of tafamidis treatment and then is identified as ‘on treatment’ until the end of observation (i.e. occurrence of death or censor date). Time‐dependent Cox proportional hazards regression analysis, using the medication as a time‐varying exposure, was performed. Hazard ratios (HRs) and the associated 95% confidence intervals (CIs) were calculated using this approach. Statistical significance was defined as *p* < 0.05 for all analyses. All statistical analyses were performed using IBM SPSS Statistics 26.0 package statistical software version 20 and Stata release 17 (Stata Corp., College Station, TX, USA).

## Results

The study population consisted of 1556 patients with ATTRwt‐CM, of whom 965 (62%) received tafamidis treatment (either meglumine 80 mg or the bioequivalent free acid 61 mg daily), primarily diagnosed from 2021 onward. The remaining 591 patients (38%), mostly diagnosed before 2021, never received disease‐modifying treatment. In the majority of cases, ATTRwt‐CM was diagnosed non‐invasively, consistent with contemporary standards. Tafamidis‐treated patients had a mean age of 78.5 ± 6.5 years, and 89.6% were men. A total of 20.8% of patients had ischaemic heart disease, 16.7% had diabetes mellitus, 67.9% had history of hypertension, and 50.6% had atrial fibrillation. Most patients were in NYHA class II (76.1%), and 17.7% and 6.2% were in NYHA class I and III, respectively. Mean maximum wall thickness in diastole (MWTd) was 17.5 ± 2.9 mm and mean left ventricular ejection fraction (LVEF) was 51.9 ± 9.7%. Most patients were in NAC disease stages 1 (63.1%) or 2 (30.1%), and 6.8% were in NAC disease stage 3. At diagnosis, beta‐blockers, angiotensin‐converting enzyme inhibitors (ACEi)/angiotensin receptor blockers (ARBs)/angiotensin receptor–neprilysin inhibitors (ARNIs) and mineralocorticoid receptor antagonists (MRAs) were prescribed in 56.3% (*n* = 543), 52.7% (*n* = 509), and 39.8% (*n* = 384) of cases, respectively. There was no significant difference between patients with and without tafamidis treatment, except for an older age, a lower NYHA class and NAC disease stage, and a greater proportion of MRA use among patients treated with tafamidis versus untreated patients. *Table* *1* shows baseline characteristics of the study population by treatment group.

### Tafamidis treatment and survival in the overall population

**Figure 2:**
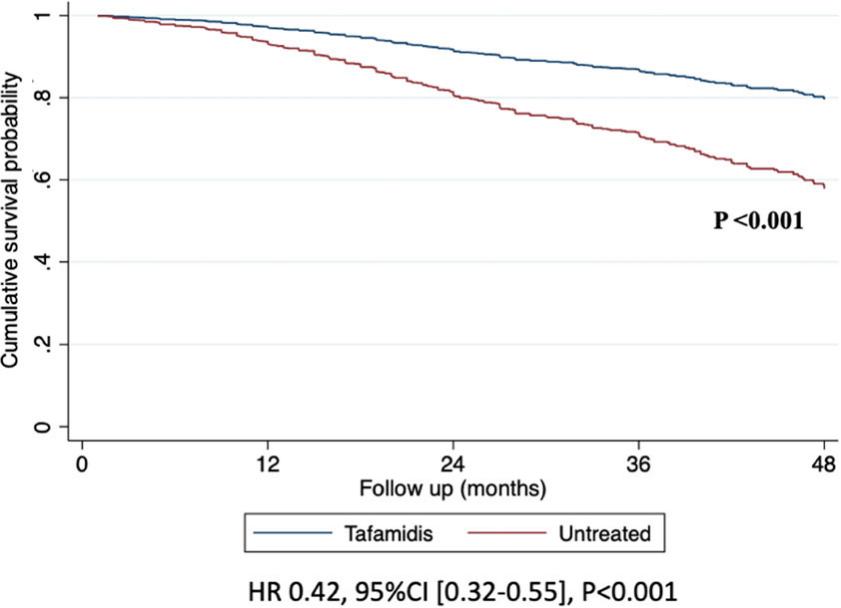
Overall survival in patients with wild‐type transthyretin amyloid cardiomyopathy according to treatment with tafamidis using a time‐dependent analysis in the total population. CI, confidence interval; HR, hazard ratio.

**Figure 3:**
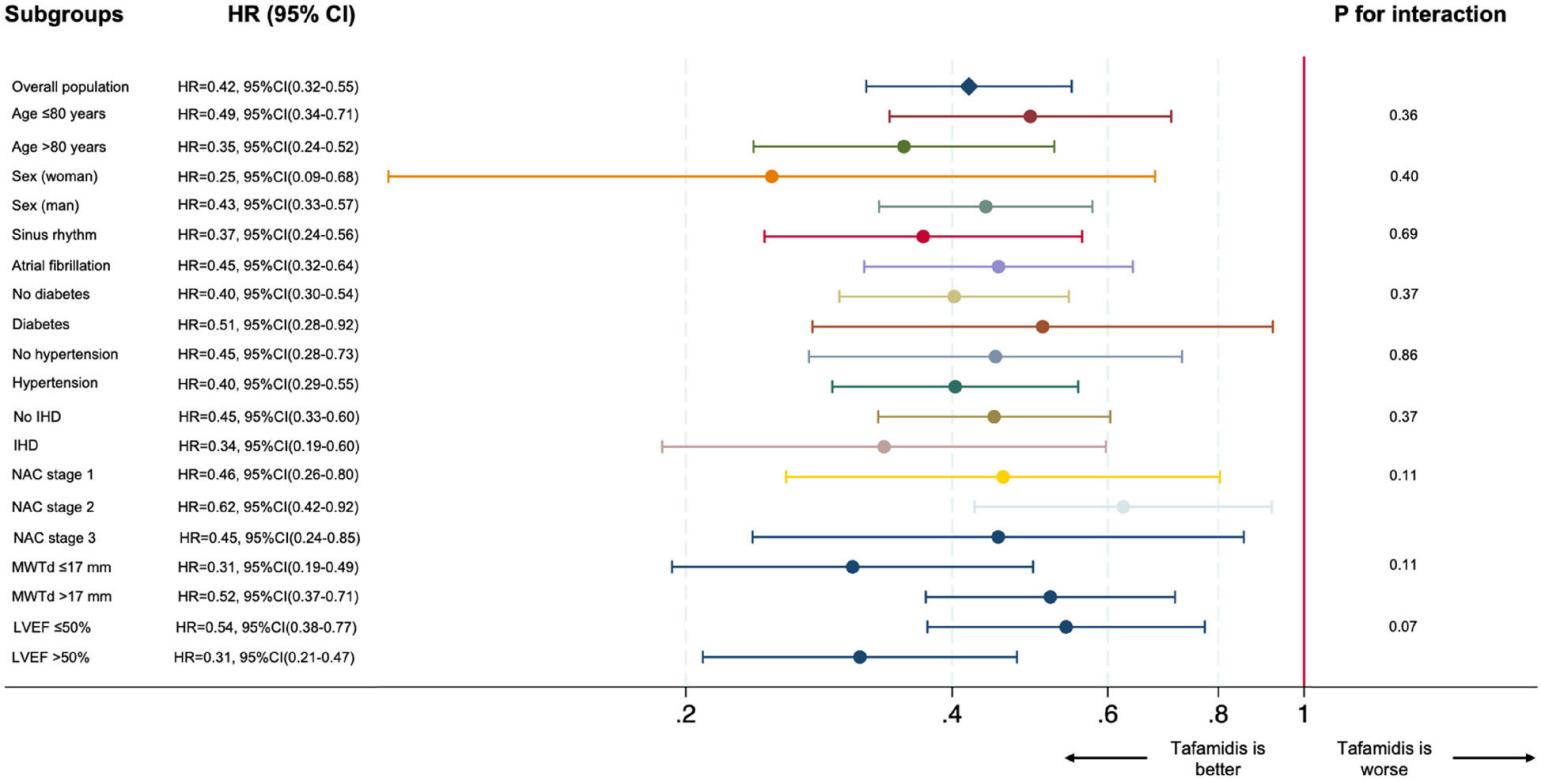
Forest plot of the association of tafamidis treatment with the risk of all‐cause mortality in patients with wild‐type transthyretin amyloid cardiomyopathy from the overall population. CI, confidence interval; HR, hazard ratio; IHD, ischaemic heart disease; LVEF, left ventricular ejection fraction; MWTd, maximal wall thickness in diastole; NAC; National Amyloidosis Centre. The first *P*‐value for interaction is the interaction between age groups, the second is the interaction between sex, the third is the interaction between heart rhythm, the fourth is the interaction between diabetes, the fifth is the interaction between hypertension, the sixth is the interaction between IHD, the seventh is the interaction between NAC disease stages, the eighth is the interaction between MWTd below and above 17 mm, and the ninth is the interaction between LVEF below and above 50%. Different groups were defined based on median and mean values of the cohort.

During a median follow‐up of 29 months (interquartile range [IQR]: 19–44), 385 (24.7%) patients died. Among the 965 patients treated with tafamidis, the death rate was 4.3 deaths per 100 patient‐years (95% CI 3.4–5.4), while, among the 591 untreated patients, the death rate was 12.5 deaths per 100 patient‐years (95% CI 11.2–14.0). Survival rates at 30 months were 89.7% (95% CI 86.6–92.1) in tafamidis‐treated patients and 75.0% (95% CI 71.6–78.0) in untreated patients. Using a time‐dependent analysis, treatment with tafamidis was associated with a lower risk of all‐cause mortality (unadjusted time‐dependent HR 0.42, 95% CI 0.32–0.55, *p* < 0.001; *Figure* *2*). The lower risk of all‐cause mortality associated with tafamidis treatment was consistent across age groups (≤80 vs. >80 years), sex, presence of atrial fibrillation, diabetes, hypertension, ischaemic heart disease, NAC disease stages, MWTd (≤17 mm vs. >17 mm), and the spectrum of LVEF (≤50% vs. >50%) (*Figure* *3*).

In a time‐dependent multivariable Cox regression analysis with covariates age, sex, ischaemic heart disease, diabetes mellitus, hypertension, atrial fibrillation, NYHA class, NAC disease stage, MWTd, LVEF, beta‐blockers, ACEi/ARBs/ARNIs, MRAs and tafamidis, only four covariates (age, higher NYHA class, higher NAC disease stage, and MWTd) were associated with a higher risk of all‐cause mortality, and only treatment with tafamidis (HR 0.56, 95% CI 0.42–0.75, *p* < 0.001) was associated with a lower risk of all‐cause mortality (*Table* *2*). For all included covariates, significant collinearity was excluded, with a variance inflation factor of 1.09.

**Table 2:** Time‐dependent multivariable Cox regression analysis for all‐cause mortality

Variables in model	Multivariable model	
	HR (95% CI)	*p*‐value
Age, years	1.06 (1.04–1.08)	**<0.001**
Male sex	1.48 (0.90–2.45)	0.12
Ischaemic heart disease	0.93 (0.68–1.26)	0.64
Diabetes mellitus	1.17 (0.86–1.58)	0.31
Hypertension	1.11 (0.84–1.47)	0.43
Atrial fibrillation	0.86 (0.67–1.10)	0.24
NYHA class		
I	Reference	Reference
II	1.61 (1.02–2.56)	**0.043**
≥III	2.55 (1.51–4.29)	**<0.001**
NAC stage		
1	Reference	Reference
2	2.29 (1.72–3.05)	**<0.001**
3	3.72 (2.54–5.45)	**<0.001**
MWTd, mm	1.04 (1.005–1.08)	**0.027**
LVEF, %	0.99 (0.98–1.004)	0.18
Beta‐blockers	0.86 (0.66–1.12)	0.27
ACEi/ARBs/ARNI	0.80 (0.62–1.03)	0.09
MRAs	1.09 (0.84–1.43)	0.51
Disease‐modifying treatment (tafamidis)	0.56 (0.42–0.75)	**<0.001**

ACEi, angiotensin‐converting enzyme inhibitor; ARB, angiotensin receptor blocker; ARNI, angiotensin receptor–neprilysin inhibitor; CI, confidence interval; HR, hazard ratio; LVEF, left ventricular ejection fraction; MRA, mineralocorticoid receptor antagonist; MWTd, maximal wall thickness in diastole; NAC, National Amyloidosis Centre; NYHA, New York Heart Association.

### Tafamidis treatment and survival in the propensity score‐matched cohort

A PS‐matched cohort was constructed to minimize the potential selection bias inherent with the treatment of tafamidis, and to assess the association between treatment with tafamidis and all‐cause mortality. The PS‐matched cohort comprised of 852 patients (426 treated with tafamidis vs. 426 not treated with tafamidis). *Table* *3* shows baseline characteristics of the PS‐matched cohort. There were no significant differences between patients with and without tafamidis treatment.

**Table 3:** Characteristics of the propensity‐score matched study population

Parameters	Tafamidis (*n* = 426)	Untreated (*n* = 426)	MSD
Age, years	78.9 ± 5.0	78.1 ± 6.6	0.100
Male sex	376 (88.3)	395 (92.7)	0.150
Atrial fibrillation	227 (53.3)	200 (46.9)	0.120
Ischaemic heart disease	103 (24.2)	78 (18.3)	0.140
Diabetes mellitus	73 (17.1)	75 (17.6)	0.012
Hypertension	294 (69.0)	281 (66.0)	0.065
Heart failure severity			
NYHA class			0.069
I	52 (12.2)	62 (14.6)	
II	319 (74.9)	309 (72.5)	
≥III	55 (12.9)	55 (12.9)	
NAC stage			0.100
1	200 (46.9)	200 (46.9)	
2	168 (39.4)	182 (42.7)	
3	58 (13.6)	44 (10.3)	
Echocardiographic parameters			
MWTd, mm	17.7 ± 2.9	17.6 ± 2.9	−0.023
LVEF, %	51.4 ± 10.2	51.3 ± 10.3	−0.005
Medications			
Beta‐blockers	240 (56.0)	243 (57.0)	0.016
ACEi/ARBs/ARNI	210 (49.3)	233 8 (54.7)	0.100
MRAs	148 (34.7)	124 (29.1)	0.132

Values are presented as mean ± standard deviation, or *n* (%).

ACEi, angiotensin‐converting enzyme inhibitor; ARB, angiotensin receptor blocker; ARNI, angiotensin receptor–neprilysin inhibitor; LVEF, left ventricular ejection fraction; MRA, mineralocorticoid receptor antagonist; MSD, mean standardized difference; MWTd, maximal wall thickness in diastole; NAC, National Amyloidosis Centre; NYHA, New York Heart Association.

**Figure 4:**
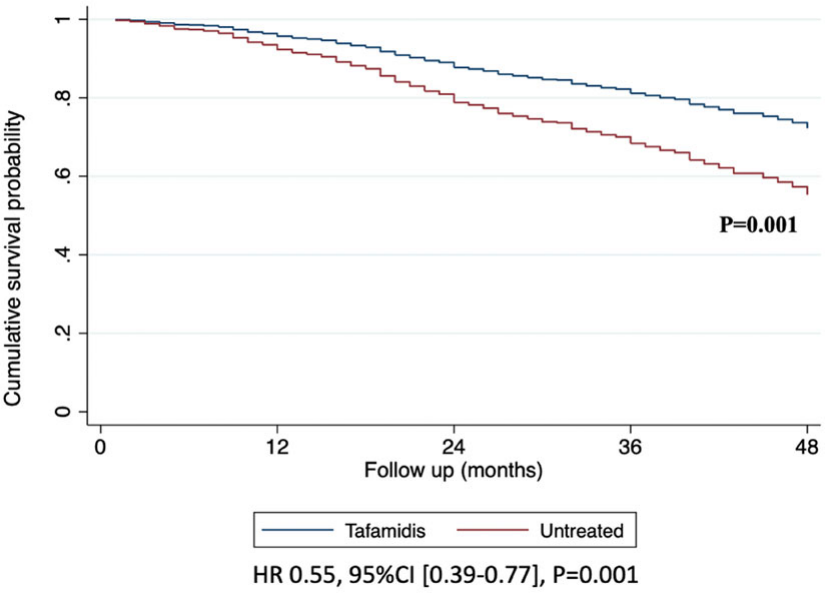
Overall survival in patients with wild‐type transthyretin amyloid cardiomyopathy according to treatment with tafamidis using a time‐dependent analysis in the propensity‐score matched population. CI, confidence interval; HR, hazard ratio.

Of the patients receiving tafamidis, 44 (10.3%) died (death rate: 6.0 deaths/100 patient‐years [95% CI 4.4–8.0]), compared with 221 (51.9%) patients not on tafamidis treatment (death rate: 14.0 deaths/100 patient‐years [95% CI 12.2–16.0]). Survival rates at 30 months were 84.9% (95% CI 78.5–89.4) in tafamidis‐treated patients and 73.3% (95% CI 69.4–77.5) in untreated patients. Among patients receiving tafamidis, median time of treatment exposure was 19 months (IQR: 15–25). Over a median follow‐up of 25 months (IQR: 15–40), treatment with tafamidis was associated with a reduced risk of all‐cause mortality using a time‐dependent analysis (HR 0.55, 95% CI 0.39–0.77, *p* = 0.001) (*Figure* *4*). These findings were consistent across the spectrum of the three NAC disease stages (*p* for interaction = 0.94).

## Discussion

In this nationwide study, which represents the largest real‐world cohort of patients with ATTRwt‐CM treated with tafamidis so far, we found that: (i) patients prescribed with tafamidis who were diagnosed in more recent years, exhibited a milder clinical phenotype and a less advanced disease stage compared to untreated patients; (ii) in the overall study population, during a median follow‐up of 29 months (IQR: 19–44), treatment with tafamidis was independently associated with lower rates of all‐cause mortality, across a broad spectrum of patient characteristics; (iii) in the PS‐matched cohort, during a median follow‐up of 25 months (IQR: 15–40), tafamidis treatment was associated with a reduced risk of all‐cause mortality across the spectrum of age, sex, comorbidities, and NAC disease stages. The unique features of this analysis lie in the inclusion of a contemporary cohort of ATTRwt‐CM patients treated with tafamidis for at least 12 months, within a network of amyloid centres operating under the same national health system of a European country.

Awareness of ATTR amyloidosis – particularly ATTR‐CM – has markedly increased over the last decade, leading to a surge in diagnoses in clinical practice. As a result, the perception of ATTR‐CM has shifted from a rare, progressive and ultimately fatal cardiomyopathy to a relatively prevalent disease with available treatments.^21^, ^22^ The ATTR‐ACT trial^7^ demonstrated the clinical benefit of tafamidis on symptoms and survival, reshaping the treatment paradigm for ATTR‐CM. As the benefit appeared less clear in patients with NYHA class III, current European guidelines^6^ recommend tafamidis for patients with wild‐type or hereditary ATTR‐CM in NYHA class I or II. In Italy, tafamidis has been approved for clinical use since October 2021 at a daily dose of 61 mg, following the same criteria. Subsequently, long‐term analyses have shown survival benefits with early initiation of tafamidis, including patients with NYHA class III.^8^, ^23^

Real‐world data on tafamidis use remain scarce, underscoring the need for further investigation as real‐world patients may significantly differ from trial participants.^11^, ^12^, ^13^, ^14^ Advances in non‐biopsy diagnosis coupled with increased awareness of ATTR‐CM among clinicians have resulted in patients being now diagnosed at earlier disease stages,^22^, ^24^ with an estimated median survival rising from 35 months in patients diagnosed before 2006 to over 60 months in recent cohorts.^25^ This pattern is confirmed in our study, where patients receiving tafamidis (mostly diagnosed from 2021 onward) exhibited a milder clinical phenotype compared to untreated patients (mostly diagnosed before 2021). Among tafamidis‐treated patients, 96% were in NYHA class I or II, and 63% were in NAC stage 1. At 30 months, survival rates in patients treated with tafamidis (89.7%) and untreated patients (75%) exceeded that reported in the treatment (70.5%) and placebo (57.1%) arms of ATTR‐ACT.^7^ Similarly, in the more recent ATTRibute‐CM,^17^ the 30‐month survival rate was 81% for patients treated with acoramidis and 74% for those in the placebo group. Our findings align with a recent real‐world analysis from THAOS, which included 587 patients treated with tafamidis.^15^ This study reported a steady increase in median survival rates among ATTR‐CM patients, with a 30‐month survival rate of 84.4% in tafamidis‐treated patients and 70% in untreated patients.^15^ Of note, the THAOS population included both wild‐type and hereditary ATTR‐CM patients diagnosed over more than 20 years, treated with variable doses of tafamidis and predominantly enrolled at US sites.^15^ In our study, to avoid the ‘immortal time’ bias, the association between tafamidis treatment and all‐cause mortality was assessed using a time‐dependent Cox regression analysis with tafamidis use as a time‐varying exposure. This approach allowed us to fully exploit the available data while avoiding bias. Treatment with tafamidis was independently associated with a lower risk of all‐cause mortality across the full spectrum of patient characteristics (*Figures* *1* and *2*). These findings were further confirmed in a time‐dependent analysis of a PS‐matched cohort, showing a 45% reduction in the risk of all‐cause mortality with tafamidis treatment. The effect sizes obtained through PS‐matched analyses are likely representing an overestimate of the true treatment effect.^26^ Although this may be partly due to the presence of unmeasured confounding bias and the relatively small number of events in the tafamidis‐treated as opposed to untreated patients (44 vs. 221 deaths), our results strongly support a benefit of tafamidis across diverse patient characteristics, with a separation of survival curves within 12 months.

Overall, these findings are highly consistent with data from ATTR‐ACT^7^ and, more broadly, ATTRibute‐CM,^17^ further demonstrating that stabilizers (tafamidis or acoramidis) do improve outcomes in real‐world patients with ATTR‐CM. With many disease‐modifying treatments available or under evaluation,^16^, ^27^, ^28^, ^29^ further studies are required to identify the optimal treatment strategy in ATTR‐CM (TTR stabilizer either alone or combined with a gene silencer or an antibody‐removal agent), tailored to disease severity and patient characteristics.

### Limitations

This study was conducted in referral centres for the diagnosis and management of ATTR amyloidosis and therefore, referral and survival bias cannot be excluded. The use of PS matching is known not to yield results that are reproduced by randomized controlled trials.^26^ The effect sizes are far larger than those reported in ATTR‐ACT and ATTRibute‐CM, likely representing an over‐estimate of the true effect size. While most tafamidis‐treated patients were diagnosed in recent years, untreated patients were primarily diagnosed before 2021. Although our findings remained significant after PS matching, we cannot exclude the possibility of other unmeasured confounders because PS matching cannot adequately adjust for all confounders. Immortal time bias is frequent in studies of this type. To avoid immortal time bias, patients entered the study from the date of diagnosis rather than the date of starting treatment, and therefore immortal time bias does not apply to our study.

The study was focused on ATTRwt‐CM, which represents a single disease entity with a more uniform pathogenic model as compared to hereditary ATTR‐CM. In addition, patients with hereditary ATTR‐CM were excluded as they are a highly heterogeneous population, often underrepresented in clinical trials in ATTR‐CM. The number of patients with ATTRwt‐CM and NYHA class III and IV was limited, particularly for those treated with tafamidis. This reflects a prescription bias in Italy, where tafamidis is restricted to patients in NYHA class I and II, excluding those in more advanced stages. Patients with NYHA class III treated with tafamidis in this analysis received treatment through a compassionate use programme. For these reasons, although NYHA class was included as a covariate in PS matching, the findings of the study are primarily representative of patients with NYHA class I and II. In addition, the study population comprised predominantly Caucasian, European patients with early‐stage ATTRwt‐CM treated within a tax‐funded universal health system. Accordingly, generalizability to populations with different ancestry, social determinants, or to private healthcare models with differential access may be limited. Due to heterogeneous, non‐adjudicated data on the cause of death across centres, sensitivity analysis on cardiovascular mortality were not performed. However, the primary outcome of the study was all‐cause mortality, the most robust outcome measure in real‐world retrospective studies.

Finally, detailed sex‐specific sub‐analyses were not feasible due to the small number of women in the study cohort, and require future dedicated analyses.

## Conclusions

In this large, real‐world, contemporary cohort of patients with ATTRwt‐CM, tafamidis‐treated patients exhibited a milder clinical phenotype and a less advanced disease stage compared to untreated patients. During follow‐up, treatment with tafamidis was consistently associated with a reduced risk of all‐cause mortality, both in the overall population and the PS‐matched cohort, across a broad spectrum of patient characteristics. Survival rates in our real‐world cohort exceeded those observed in the ATTR‐ACT trial, further confirming the importance of early diagnosis and initiation of disease‐modifying treatment in this patient population.

### Funding

For ‘Cardiology Unit, Cardiac Thoracic and Vascular Department, IRCCS Azienda Ospedaliero‐Universitaria di Bologna, Bologna, Italy’, the work reported in this publication was funded by the Italian Ministry of Health, RC‐2024‐2 789 983 project. No funding was received for other participating centres.

**Conflict of interest**: A.P. received fees for education activities from Alnylam and honoraria from Bayer outside the submitted work. P.M. received speaker fees from Jansen, Sebia, Pfizer (also research grant), Prothena and served on advisory board for Siemens. S.L. has consulting income from Alnylam and Pfizer. F.C. has consulting income from Alnylam, Pfizer, AstraZeneca, Bridgebio, Novo Nordisk, Amicus, Daiichi Sankyo and Bayer. A.C. has received consulting income from AstraZeneca, and has advisory role for Pfizer, AstraZeneca and Bayer. D.T. received speaker fees from Alnylam, AstraZeneca, Boehringer Ingelheim, and Pfizer. M.C. received speaker and advisor fees in the last 2 years from Akcea Therapeutics, Alnylam, AstraZeneca, Bristol Myers Squibb, Novartis, Pfizer, Sanofi Genzyme and two investigator‐initiated grants from Pfizer, without any control on intellectual contents. F.P. received speaker fees from Alnylam and Pfizer. G.L. received consulting income and/or unrestricted grants from Pfizer, AstraZeneca, Bayer. M. Metra received consultant honoraria of minimal amount from AstraZeneca, Bayer, Boehringer Ingelheim, Novo Nordisk, Roche diagnostics in the last 3 years. S.P. received speaker and advisor fees from Akcea Therapeutics, Alnylam and Pfizer. M. Merlo received fees from Pfizer, Novartis and Vifor Pharma, and an unrestricted general research grant on amyloidosis by Pfizer, without any control on intellectual contents. G.P. received honoraria from Alexion, Abbvie, Bayer, Life Molecular Science, Protego, Pfizer, Prothena, Regeneron. G.S. received fees from Biotronik, Boston Scientific, AstraZeneca and Novartis. All other authors have nothing to disclose.

## Supporting information


**Appendix S1.** Supporting Information.
