# Supplementary material for: Clinical phenotype and prognosis of real‐world patients with wild‐type transthyretin amyloid cardiomyopathy treated with tafamidis
Source: Eur J Heart Fail. 2025 Nov 29;27(12):2952–60. doi: 10.1002/ejhf.70071 (PMC12803573; doi:10.1002/ejhf.70071)
Supplement: Supplementary file 1 — Appendix S1. Supporting Information. [file EJHF-27-2952-s001.docx]

**SUPPLEMENTARY MATERIAL**

**Supplementary Material**

**Echocardiographic methods**

At least 3 consecutive beats were recorded for each view, and images were stored for off-line analysis. LV chamber morphology was assessed following the latest American Society of Echocardiography/European Association of Cardiovascular Imaging Guideline. LV ejection fraction (EF) was calculated with the biplane Simpson’s method from volumes acquired in both the 4-chamber and the 2-chamber views.”

**Supplementary Table 1. Participating centres across Italy**

| **Center** |
| --- |
| Pavia |
| Bologna |
| Firenze |
| Ancona |
| Pisa |
| Padova |
| Forlì |
| Lecce |
| Napoli |
| Roma |
| Ferrara |
| Milano |
| Brescia |
| Trieste |
| Genova |
| Pescara |
| Bergamo |
| Messina |
| Udine |
